# Heme acts as a metabolic brake on erebosis in the *Drosophila* gut

**DOI:** 10.1371/journal.pbio.3003942

**Published:** 2026-08-19

**Authors:** Motohiro Morikawa, Tetsutaro Hayashi, Yuko Ikegawa, Tomomi Takano, Kazuya Wata, Hirohisa Kyogoku, Mariko Kuse, Mika Yoshimura, Hiroshi Nishida, Rahul Parit, Dao My Linh, Kanta Kawai, Tasuku Hirayama, Chun Wang Sy, Kan Etoh, Itoshi Nikaido, Sa Kan Yoo

**Affiliations:** 1 Division of Developmental Biology and Regenerative Medicine, Kobe University, Kobe, Japan; 2 Laboratory for Homeodynamics, RIKEN Center for Biosystems Dynamics Research (BDR), Kobe, Japan; 3 Omics AI Research Team, Advanced General Intelligence in Science Program (AGIS), TRIP Headquarters, RIKEN, Wako, Japan; 4 Graduate School of Agricultural Science, Kobe University, Kobe, Japan; 5 Laboratory for Chromosome Segregation, RIKEN Center for Biosystems Dynamics Research (BDR), Kobe, Japan; 6 Laboratory of Chemical Biology, Gifu Pharmaceutical University, Gifu, Japan; 7 Faculty of Science, University of Hong Kong, Hong Kong, China; 8 Department of Molecular Cell Biology, Graduate School of Medical Sciences, Kyushu University, Fukuoka, Japan; 9 Department of Functional Genome Informatics, Division of Biological Data Science, Medical Research Laboratory, Institute of Integrated Research (IIR), Institute of Science Tokyo, Tokyo, Japan; Universiteit Gent, BELGIUM

## Abstract

Tissue homeostasis relies on the balance between proliferation of stem cells and death of differentiated cells. In *Drosophila* gut enterocytes, we recently identified a novel form of cell death, termed erebosis. Erebosis is a nonapoptotic, nonautophagic, and nonnecrotic process, in which affected cells accumulate Ance (angiotensin-converting enzyme) and lose many other proteins, ultimately leading to the loss of organelles and the nucleus. The underlying molecular mechanism of erebosis has remained unclear. Here, through single-cell RNA sequencing and genetic approaches, we found that the small metabolite heme regulates erebosis. Cells undergoing erebosis up-regulate the heme-degrading enzyme *Heme oxygenase (Ho)* and the heme exporter *Mrp5*, and decrease intracellular amounts of heme. Heme depletion by *Mrp5* overexpression promotes erebosis, whereas heme accumulation by knockdown of *Ho* or *Mrp5*, or by feeding a heme precursor, suppresses it. Downstream of heme, Dpp signaling suppresses erebosis. Inhibition of erebosis reduces intestinal stem cell proliferation, indicating a cross-talk mechanism between enterocyte death and stem cell division. Our results demonstrate that reduction of cytoplasmic heme is a critical step in initiating enterocyte erebosis and coordinating stem cell proliferation, thereby maintaining gut tissue homeostasis. This work provides the first insight into the molecular mechanism regulating erebosis.

## Abstract 2

Erebosis is a non-apoptotic, non-autophagic and non-necrotic process in the Drosophila gut, in which affected cells accumulate angiotensin-converting enzyme. This study shows that the decrease of cytoplasmic heme is a critical step in initiating enterocyte erebosis and coordinating cell proliferation to maintain tissue homeostasis.

## Introduction

Dynamic tissue homeostasis of the gut is maintained by cell turnover, which is mediated by intestinal stem cells (ISCs) and enterocyte death. Since the discovery of ISCs in the *Drosophila* gut [1,2], there has been intensive research on the regulatory mechanisms controlling ISC activity [3–11]. Regarding enterocyte death, many studies have used experimental conditions that artificially induce apoptosis or necrosis, such as exposure to infection, DSS, paraquat, or bleomycin [12–15]. Genetic inhibition of genes that are essential for tissue maintenance, such as Rab21 or septate junction components, can trigger enterocyte apoptosis [16,17]. Inhibition of the unconventional myosin, Myo1D, also disturbs physiological function of enterocytes [18]. However, our understanding of how enterocyte death is regulated under normal physiological conditions remains very limited.

We recently demonstrated that gut cell death under the homeostatic condition is predominantly erebosis, a previously unrecognized mode of cell elimination in adult tissue homeostasis [19]. Erebosis is a distinct form of cell death that is nonapoptotic, nonautophagic, and nonnecrotic [20], which most frequently occurs in the R4 region. A recent report also demonstrates that enterocyte turnover is regulated in an apoptosis-independent manner [21], consistent with the notion that nonapoptotic cell elimination such as erebosis contributes to gut homeostasis. Interestingly, erebosis was suggested to exist in the mammalian brain [22]. Erebotic cells accumulate Ance (angiotensin-converting enzyme) while losing many other intracellular proteins, ultimately leading to the loss of organelles and the nucleus [19]. Despite phenotypic characterization of erebosis, its molecular mechanism remains to be solved. In this study, we sought to elucidate the molecular basis underlying erebosis.

## Results

To investigate the mechanism of erebosis, we decided to perform single-cell RNA sequencing. We found that, following the cell dissociation process using trypsin or elastase, it was difficult to isolate erebotic cells. In addition, the tissue dissociation procedure, which lasts at least 1 hour at 37 °C, can induce stresses and alter stress-response-related gene expression [23]. We thus opted to use micromanipulation under a microscope to collect live cells directly from the gut, immediately lyse the collected cells, and perform RamDA-seq, a highly sensitive single-cell total RNA sequencing method [24].

Erebotic cells in live guts can be identified using the GFP/nlsRFP reporter [19]. In enterocytes expressing GFP and nlsRFP, GFP disappearance occurs concomitantly with Ance accumulation during erebosis, which is followed by the later loss of nlsRFP [19]. Especially, GFP negativity with preserved nlsRFP fluorescence marks early-to-intermediate erebosis, whereas loss of both GFP and nlsRFP represents either late-stage erebosis [19] or necrosis, during which both cytoplasmic and nuclear proteins are lost [25–27]. Apoptotic cells, which often become apically extruded, do not lose fluorescent proteins [16,17,28,29]. To roughly categorize cells as erebotic or normal based on their fluorescence, we expressed GFP and nlsRFP in enterocytes using the *Myo1D-Gal4* driver. We picked up GFP− cells with intact nlsRFP fluorescence, which we phenotypically classified as erebotic but neither necrotic nor apoptotic. By taking advantage of the GFP/nlsRFP signals, we aimed to collect approximately equal numbers of normal enterocytes and erebotic cells.

We collected 8 GFP+ normal enterocytes and 8 GFP− erebotic cells from the R4 region [30], a region where erebosis readily occurs, of two guts, and performed RamDA-seq (S1A Fig). Of the 16 cells, only one failed the quality control analysis, and the remaining 15 were subjected to further analyses (S1B–S1D Fig). Despite the limited number of cells, we obtained high-quality data (GSE308217) in which enterocytes were grouped into three clusters (S1E Fig). Based on GFP fluorescence patterns and gene ontology (GO) analysis, we interpreted the three clusters as a normal cell cluster, an erebotic cluster, and a damaged cell cluster (S1E Fig). Classification of the normal cell cluster was straightforward because all enterocytes in this cluster were GFP+. The cluster we categorized as the erebotic cell cluster included both GFP+ and GFP− cells, suggesting that changes in gene expression patterns precede phenotypic manifestation such as GFP loss. In support of this interpretation, in situ hybridization revealed that GFP− erebotic cells expressed CG8745 and LManIII, which were specifically upregulated in this erebotic cell cluster (S2A, S2B Fig). In contrast, GFP+ normal enterocytes expressed Srg3/CG33926, which was upregulated in the normal cell cluster (S2A, S2B Fig). We also validated the classification with Fly Cell Atlas dataset [31] using module score analysis [32]. Module score of R4-specific genes [30] identified “Enterocyte of posterior adult midgut epithelium” and “Midgut large flat cell” clusters as R4 enterocytes in the Fly Cell Atlas data (S3A–S3C Fig). Module scores of top 20 genes in the normal cell cluster or the erebotic cell cluster (S4 Fig) were enriched in different populations of R4 enterocytes (S3D, S3E, S3G Fig), indicating that the cell population identified in our analysis exists distinctly in Fly Cell Atlas dataset. The cluster we categorized as the damaged cell cluster, composed entirely of GFP− cells, showed high activation of the Toll, IMD, and JAK/STAT pathways in the GO analysis (S2C Fig). These pathways are known to be activated by infection and tissue injuries [14,33], implying that this cluster was under such stresses. Consistently, cells in this cluster were isolated at the end of the collecting procedure, exposed to the nonphysiological environment for longer time. Moreover, we previously showed that the IMD pathway or Upd2 is not involved in erebosis [19]. Artificial activation of STAT using the *Myo1D-Gal4* driver with *tub-Gal80*^*ts*^ (referred to as *Myo1D-Gal4*^*ts*^) did not induce erebosis either (S2D Fig). Furthermore, Fly Cell Atlas data did not have a distinct cell population with an enrichment of top20 genes of the damaged cell cluster (S3F Fig). Thus, we assume that this cluster is not related to erebosis. However, we cannot completely exclude the possibility that it may represent a very late stage of erebosis, where gene expression patterns may become dysregulated.

Initially, we became interested in genes whose expression was altered in the erebotic cell cluster. We tested RNAi-mediated knockdown of differentially expressed genes (DEGs) in the erebotic cluster, such as CG8745 (S4 Fig). However, none of them affected erebosis. We speculate that many of the genes upregulated in this cluster are involved in the processes that occur after initiation of erebosis, but not in its initiation.

Next, we turned our attention to the normal cell cluster, hypothesizing that it might include “pre-erebosis” cells. GO analysis revealed strong enrichment of the proteasome degradation pathway in this cluster (S5A Fig). Further examination showed that three cells in this cluster particularly upregulated nearly all proteasome genes (S5B Fig). We also confirmed that proteasome inhibition suppresses erebosis, accumulating polyubiquitinated proteins (S5C Fig). We note that the effects of proteasome inhibition should be interpreted with caution, as the proteasome regulates a wide range of biological processes. For example, proteasome inhibition can induce apoptosis by preventing DIAP1-mediated degradation of caspases [34,35]. Thus, to better define the relevant mechanism, it was important to manipulate specific pathways correlated with proteasome activation. For that purpose, we decided to screen DEGs in the proteasome-high cells, which we interpreted as putative pre-erebotic cells.

**Fig 1:**
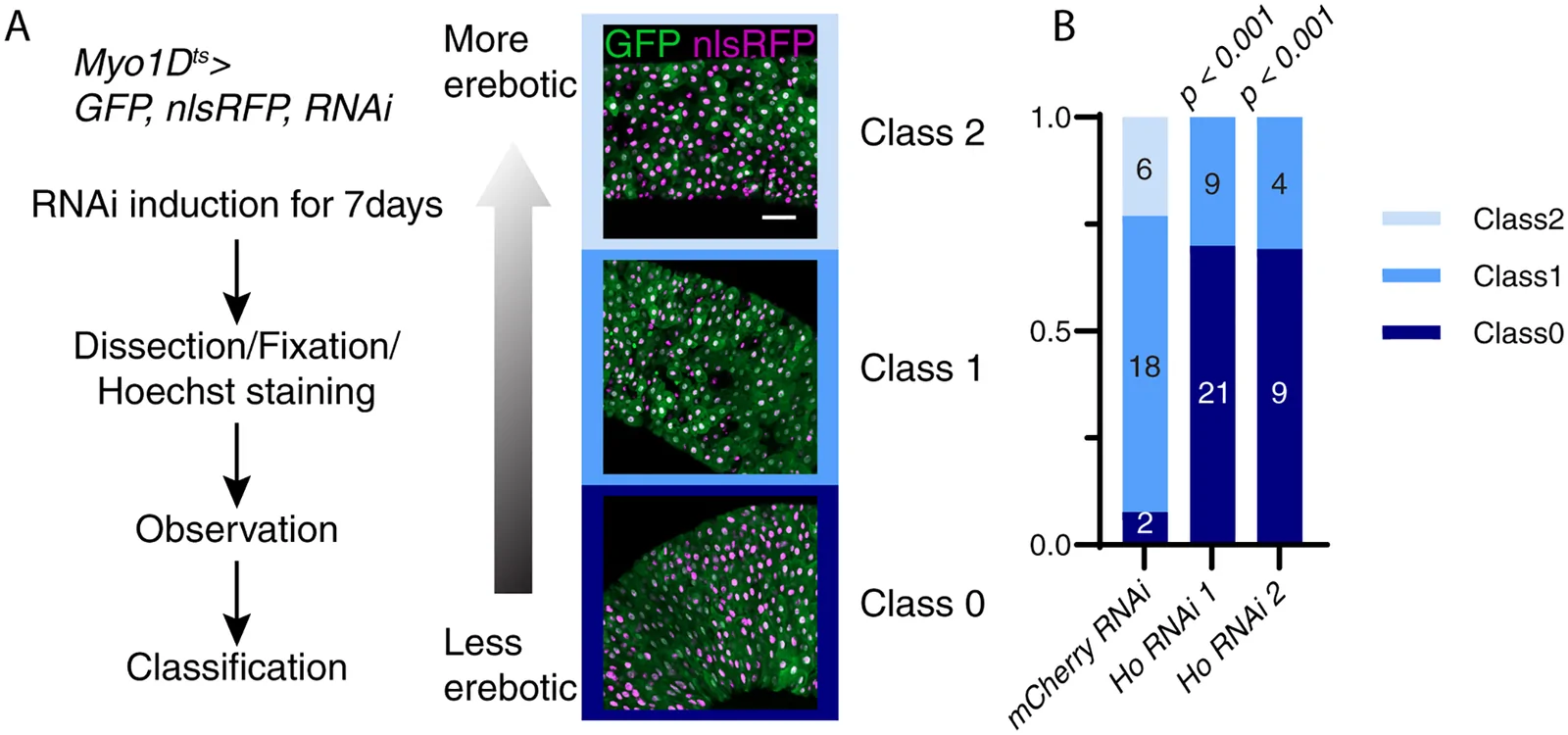
RNAi screening identified *Heme oxygenase* as an erebosis regulator. **(A)** Schematic illustration of RNAi screening and classification of erebosis. After RNAi induction at 30 °C for 7 days, the guts were dissected, fixed, and stained with Hoechst 33342, followed by observation with a confocal microscope. The extent of erebosis was classified based on the number of GFP− nlsRFP+ erebotic cells: class0- three or fewer erebotic cells/view, class1- intermediate between class0 and 2, class2- > 30% erebotic cells/view. Scale bar, 50 μm. **(B)** Multiple RNAi stocks of *heme oxygenase* (*Ho*) inhibit erebosis. *P* values were calculated using Fisher’s exact test and adjusted with the Benjamini–Hochberg method (B).

For the screening, we focused on the number of GFP− nlsRFP+ enterocytes, as this state represents the most frequent early/intermediate stage of erebosis. We classified the extent of erebosis into three groups based on the number of erebotic cells per microscopic field (Fig 1A): class 0 – three or fewer erebotic cells; class 1 – between class 0 and 2; and class 2 – many (>30% of all enterocytes) erebotic cells.

As a result of the screening, we discovered that *Heme oxygenase* (*Ho*) *RNAis* reduced erebosis (Fig 1B). In guts expressing *Ho RNAis*, more than 50% were classified as class 0, whereas the control expressing *mCherry RNAi* showed less than 30% class 0 guts. Two independent RNAi stocks similarly reduced erebosis, indicating that the reduction is unlikely to be due to the off-target effect of RNAi. Knockdown efficiency was also confirmed by measuring mRNA levels (S6A Fig). Consistent with the design of the screening, *Ho* is upregulated in the pre-erebosis cells with proteasome enrichment (S5B Fig).

Ho is an enzyme that degrades heme, an iron-containing tetrapyrrole that plays essential roles in diverse biological functions, including oxygen transport, oxidative metabolism, and xenobiotic detoxification [36]. Heme can function by binding to proteins or, in its labile form, as an oxidant itself. Its degradation produces oxidative Fe^2+^ iron, as well as the antioxidants, bilirubin and carbon monoxide (CO).

**Fig 2:**
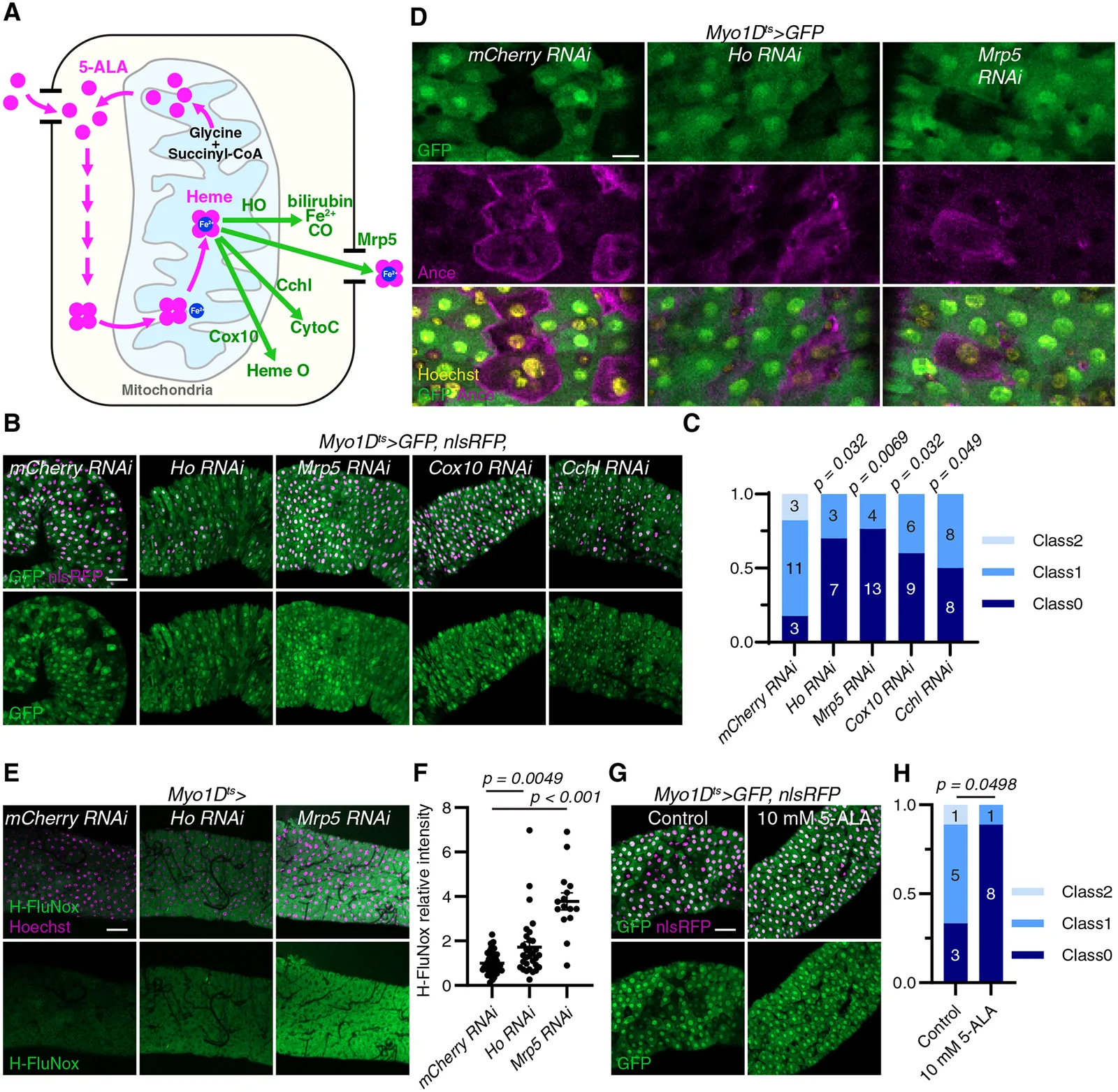
Excess heme inhibits erebosis. **(A)** Illustration of the heme synthesis and metabolism pathway. **(B)** Knockdown of heme degradation, export, or conversion genes (*Ho*, *Mrp5*, *Cox10*, *Cchl*) tends to reduce GFP− nlsRFP+ erebotic cells compared to control *mCherry RNAi*. Scale bar, 50 μm. **(C)** Classification of (B). RNAi of *Ho*, *Mrp5*, *Cox10*, or *Cchl* leads to 50% or more class0 guts and no class2, while in control, class0 is lower than 50%. **(D)** Anti-Ance antibody stains GFP− erebotic cells with *Ho RNAi* and *Mrp5 RNAi*, as well as control *mCherry RNAi* guts. Scale bar, 10 μm. **(E)** A fluorescent heme probe H-FluNox shows that *Ho RNAi* and *Mrp5 RNAi* increase intracellular heme. Scale bar, 50 μm. **(F)** Quantification of (E). Both of *Ho RNAi* and *Mrp5* RNAi significantly increase H-FluNox signal. **(G)** Feeding of 10 mM 5-ALA reduces erebosis compared with control. Scale bar, 50 μm. **(H)** Classification of (G). 5-ALA feeding results in >50% class0 guts. *P* values were calculated using Fisher’s exact test (C, H) with Benjamini–Hochberg adjustment (C) or using Dunnett’s test (F). S1 Data provides the data shown in (F).

Since Ho inhibition can affect many metabolites, including heme, bilirubin, Fe^2+^, and CO, the next step was to clarify which molecule regulates erebosis. We decided to test the effects of manipulating heme metabolism on erebosis. Heme is synthesized from glycine and succinyl-CoA via 5-aminolevulinic acid (5-ALA) [37](Fig 2A). It is degraded by Ho or further converted by Cox10 or Cchl [38–40]. In *Drosophila*, heme is exported out of the cell by Mrp5 [41]. We performed RNAi-mediated knockdown of genes involved in heme conversion or export. Inhibition of heme export with *Mrp5 RNAi* markedly reduced erebosis, while inhibition of heme conversion (*Cox10, Cchl RNAi*) showed only a mild trend of suppression (Fig 2B, 2C). As with *Ho RNAi*, these manipulations are expected to increase intracellular heme, indicating that heme itself, rather than its metabolites, influences erebosis. The stronger effects of *Ho* or *Mrp5* suppression compared to conversion inhibition suggest that heme degradation and export are key processes limiting intracellular heme. Notably, *Mrp5* is upregulated in pre-erebotic cells (S5B Fig), similar to *Ho*, which would be expected to further decrease the cytoplasmic heme. We confirmed that even under the conditions with reduced erebosis following RNAi treatment, GFP and Ance inversely correlate (Fig 2D), indicating that the persistence of GFP signals is not an artifactual effect and that heme controls erebosis.

We investigated whether *Ho RNAi* or *Mrp5 RNAi*, both of which achieved efficient gene knockdown (S6A, S6B Fig), truly affects intracellular heme levels. We visualized intracellular heme using the fluorescent heme sensor H-FluNox, which becomes fluorescent upon binding to heme in a live condition [42]. Imaging with H-FluNox revealed that these anti-erebotic RNAis increased intracellular heme levels in enterocytes (Fig 2E, 2F).

Besides the genetic approaches, we also increased the intracellular amount of heme nongenetically. Feeding of a heme precursor 5-ALA, which is known to elevate intracellular heme [42], also reduced erebosis (Fig 2G, 2H). From these results, we conclude that heme itself, rather than its metabolites, suppresses erebosis.

**Fig 3:**
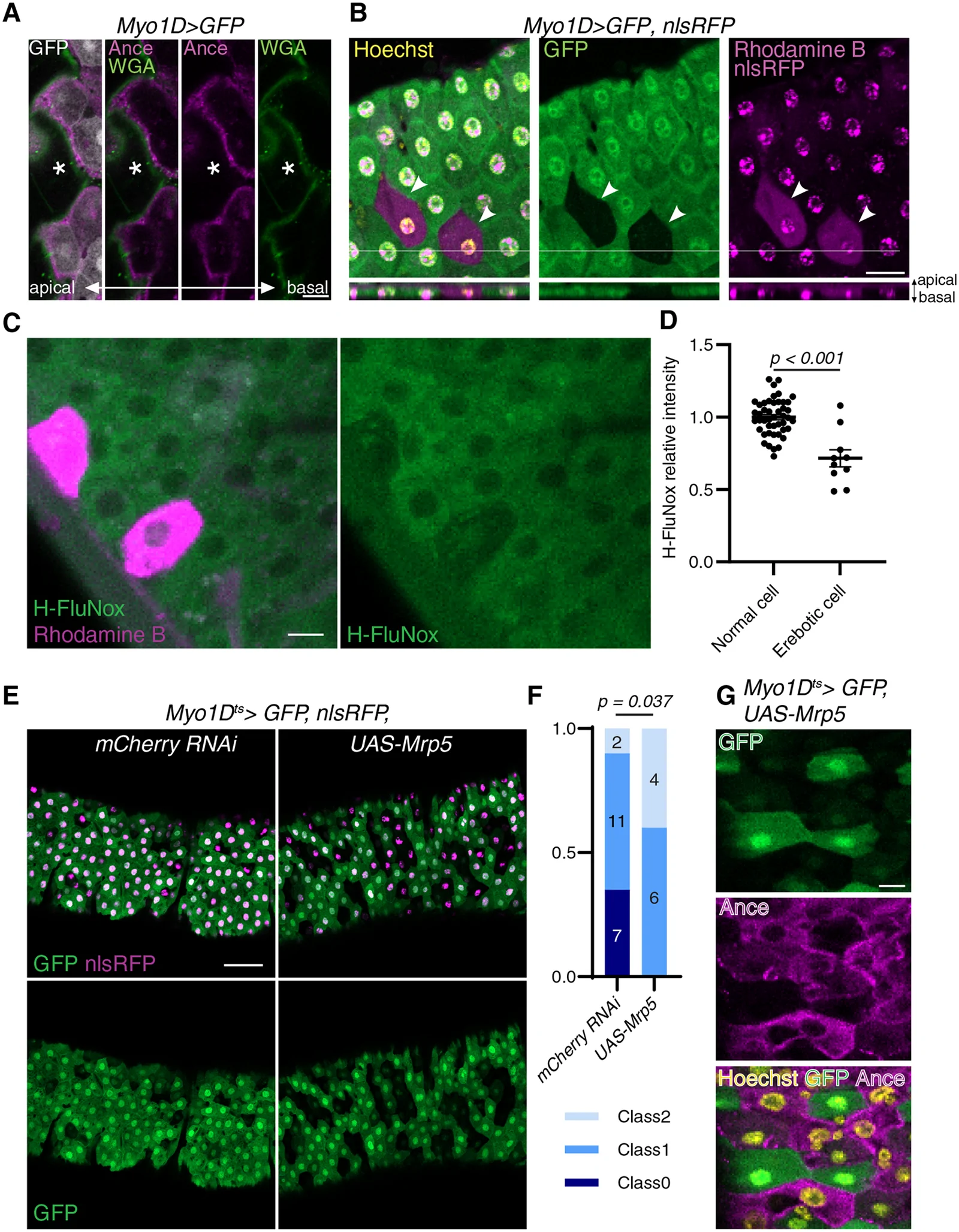
Depletion of heme triggers erebosis. **(A)** Ance (magenta) localizes just above enterocytes (GFP, white) and below the peritrophic membrane (WGA, green). White asterisks indicate the gut lumen. Scale bar, 10 μm. **(B)** GFP− erebotic cells (arrowheads) become Rhodamine+. The bottom pictures show Z projection of GFP+ normal enterocytes and GFP low erebotic cells. Scale bar, 20 μm. **(C)** Heme is depleted in erebotic cells. To measure heme levels, flies were fed with erebosis-suppressive 1x SY food for 4 days, followed by erebosis-inducing Rhodamine food for 1 day. Dissected guts were stained with a heme probe H-FluNox. In Rhodamine+ erebotic cells, H-FluNox (green) is decreased. Scale bar, 10 μm. **(D)** Quantification of (C). H-FluNox signal is significantly reduced in erebotic cells compared to surrounding normal enterocytes. **(E)** Overexpression of a heme exporter *Mrp5* induces erebosis. Scale bar, 50 μm. **(F)** Classification of (E). *Mrp5* overexpression leads to 40% class2 and no class0, while control *mCherry RNAi* results in >15% class2 and >20% class0. **(G)** Ance exclusively localizes to GFP− erebotic cells even with *Mrp5* overexpression. Scale bar, 10 μm. *P* values were calculated using unpaired two-tailed *t* tes*t* (D) or Fisher’s exact test (F). S1 Data provides the data shown in (D).

Transcriptomic analysis revealed that *Ho* is upregulated in pre-erebosis cells, suggesting that heme is decreased in the initiation process of erebosis. To investigate this possibility, we examined heme levels in enterocytes using the heme probe H-FluNox. Since signals of H-FluNox and GFP overlap, we sought for an alternative method to label erebotic cells. Although erebosis is distinct from necrosis, it is characterized by the acute and simultaneous occurrence of both cellular entry of extracellular Ance, which is normally located between enterocytes and the peritrophic membrane (Fig 3A), and loss of cytoplasmic proteins [19], due to transient membrane pore formation [43]. Feeding flies with Rhodamine B, a red fluorescent dye, prior to imaging enables selective labeling of erebotic cells (Fig 3B) [43]. Combining Rhodamine B-mediated pre-labeling of erebotic cells and heme imaging with H-FluNox, which was added at the time of imaging, we found that H-FluNox signals were weaker in Rhodamine+ erebotic cells compared to neighboring Rhodamine- normal enterocytes (Fig 3C). Quantitative analysis confirmed that erebotic cells significantly reduced H-FluNox signals, indicating that intracellular heme is reduced in these cells (Fig 3D).

To further test whether heme reduction induces erebosis, we artificially decreased intracellular heme in enterocytes by ectopic expression of Mrp5, a heme exporter. This manipulation led to an increase in erebosis (Fig 3E, 3F). GFP− erebotic cells exclusively accumulated Ance, again revealing that heme is upstream of GFP clearance and Ance incorporation (Fig 3G). These results indicate that the depletion of heme is a critical step for cells to initiate erebosis. Importantly, p35 expression did not reverse the effects of heme manipulation on erebosis (S6C, S6D Fig), indicating that these effects are not mediated by apoptosis.

**Fig 4:**
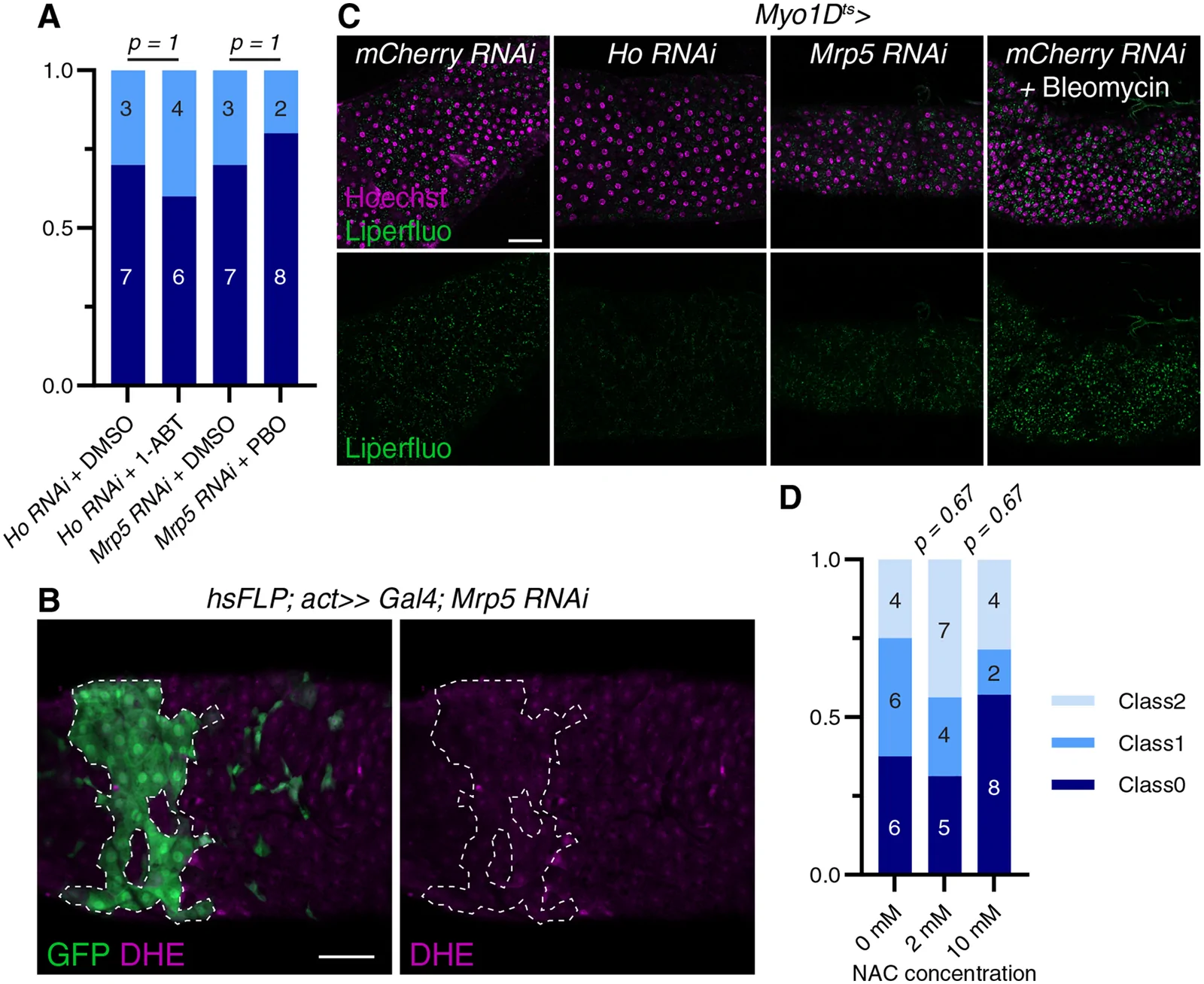
Heme inhibits erebosis independent of CYP P450 or ROS. **(A)** Feeding of CYP P450 inhibitors 1-Aminobenzotriazole (1-ABT) or piperonylbutoxide (PBO) does not restore the inhibition of erebosis by heme. 1-ABT with *Ho RNAi* and PBO with *Mrp5 RNAi* result in >50% class0 and no class2, which is similar to *Ho RNAi* or *Mrp5 RNAi* with DMSO. **(B)** ROS levels are not increased by *Mrp5 RNAi*. Using hsFLP system, *Mrp5 RNAi* was induced only in GFP+ cells. ROS probe dihydroethidium (DHE, magenta) is not changed in GFP+ *Mrp5 RNAi* cells (dashed line). **(C)** *Ho RNAi* or *Mrp5 RNAi* does not increase signals of lipid peroxide probe Liperfluo (green), while bleomycin increases them. **(D)** Feeding of a reducing agent N-acetylcysteine (NAC) does not increase erebosis. *P* values were calculated using Fisher’s exact test (A, D) and adjusted with the Benjamini–Hochberg method (D). Scale bars, 50 μm.

Next, we explored how intracellular heme inhibits erebosis. Heme is known to serve multiple functions, such as acting as a coenzyme for cytochrome P450s and contributing to the generation of reactive oxygen species (ROS) [36]. Inhibition of heme oxygenase could potentially induce oxidative stress due to both the accumulation of oxidative labile heme and the decrease in reducing bilirubin. Since there are at least 85 cytochrome P450s in *Drosophila* [44], genetic knockdown may not be productive due to redundancy. Thus, we resorted to pharmacological approaches to inhibit cytochrome P450s. We treated flies with cytochrome P450 inhibitors 1-Aminobenzotriazole or Piperonyl butoxide, both of which are potent cytochrome p450 inhibitors and have been used in *Drosophila* adults [45,46]. Neither of them restored erebosis in enterocytes that expressed *Ho RNAi* or *Mrp5 RNAi* (Fig 4A), suggesting that cytochrome P450s are not responsible for heme-mediated regulation of erebosis. Next, we tested whether excess heme promotes ROS generation in the *Drosophila* midgut. Using dihydroethidium (DHE), we measured ROS levels in *Mrp5 RNAi* clones generated by the heat shock-induced flippase system, which enables sensitive detection of ROS levels in comparison to surrounding wildtype enterocytes. DHE levels were not changed between *Mrp5 RNAi* clones and surrounding control cells (Fig 4B). Additionally, a lipid peroxide marker Liperfluo signal was not increased by *Ho RNAi* or *Mrp5 RNAi*, whereas it was elevated by the positive control, bleomycin treatment (Fig 4C). Furthermore, feeding antioxidant N-acetylcysteine did not suppress erebosis (Fig 4D). Taken together, these results indicate that heme inhibits erebosis independent of cytochrome P450 activity or ROS generation.

**Fig 5:**
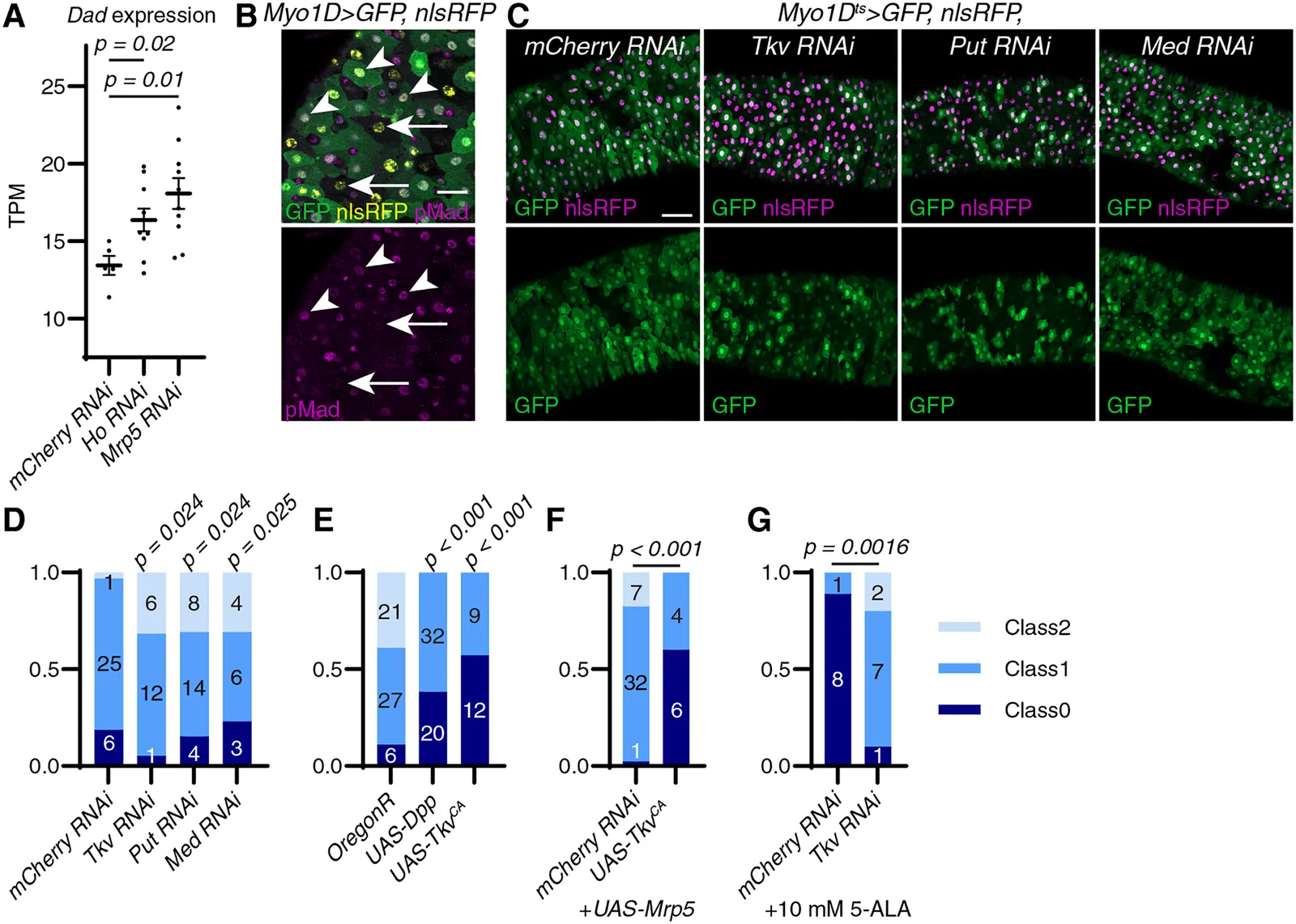
Dpp signaling mediates erebosis downstream of heme. **(A)** Erebosis reduction upregulates a Dpp marker *Dad*. Bulk RNA-seq of the whole gut indicates that *Ho RNAi* and *Mrp5 RNAi* increase the expression of a Dpp signaling marker gene *Dad*. **(B)** Erebotic cells reduce a Dpp marker phosphorylated Mad (pMad). Immunostaining of pMad (magenta) is reduced in GFP− erebotic cells (arrows) compared with GFP+ normal enterocytes (arrowheads). Scale bar, 25 μm. **(C)** Dpp inhibition increases erebosis. Suppression of Dpp signaling by RNAi of *Thickveins* (*Tkv*), *Punt* (*Put*), or *Medea* (*Med*) increases GFP− nlsRFP+ erebotic cells compared with control *mCherry RNAi*. Scale bar, 50 μm. **(D)** Classification of (C). *Tkv RNAi*, *Put RNAi*, and *Med RNAi* significantly increase erebosis. **(E)** Dpp activation inhibits erebosis. Classification reveals that overexpression of *Dpp* and a constitutively active mutant of *Tkv* (*Tkv*^*CA*^) significantly reduce erebosis. **(F)** Dpp activation restores the erebosis induction by heme depletion. Co-expression of *Tkv*^*CA*^ significantly inhibits erebosis induced by *UAS-Mrp5*. **(G)** Dpp inhibition reverses the erebosis inhibition by heme accumulation. *Tkv RNAi* significantly increases erebosis under the erebosis suppression by 5-ALA feeding. *P*-values were calculated using likelihood ratio test (A) or Fisher’s exact test (D–G) and adjusted with the Benjamini–Hochberg method (A, D, E). S1 Data provides the data shown in (A).

To further explore molecular mechanisms by which heme suppresses erebosis, we employed RNA-seq analysis. We analyzed guts from two different RNAi lines for each of *Ho RNAi* and *Mrp5 RNAi* to minimize the off-target effects of RNAis. We discovered that both of *Ho RNAi* and *Mrp5 RNAi* upregulated *Dad*, a Dpp signaling marker gene [47] (Fig 5A). We also observed a modest trend toward increased expression of the Dpp ligands, Dpp and Gbb (S7A Fig). Dpp signaling regulates gut tissue homeostasis via its regulation of ISCs and enterocytes [8,48–51]. Consistent with the transcription data, erebotic cells showed weaker phosphorylated Mad signals (Fig 5B), indicating that Dpp signaling is suppressed during erebosis. RNAis for Dpp signaling components *Tkv*, *Put*, and *Med* increased erebosis (Fig 5C, 5D), while Dpp activation by *Dpp* or the constitutively active form of *Tkv* reduced it (Fig 5E). Moreover, Dpp activation reversed erebosis induction by *Mrp5* overexpression, while Dpp inhibition suppressed erebosis inhibition by feeding of the heme precursor 5-ALA (Fig 5F, 5G). Dpp-mediated effects on erebosis were not suppressed by p35 (S7B, S7C Fig), indicating that the effects are independent of caspase activation. Taken together, heme positively regulates Dpp signaling, which in turn suppresses erebosis.

**Fig 6:**
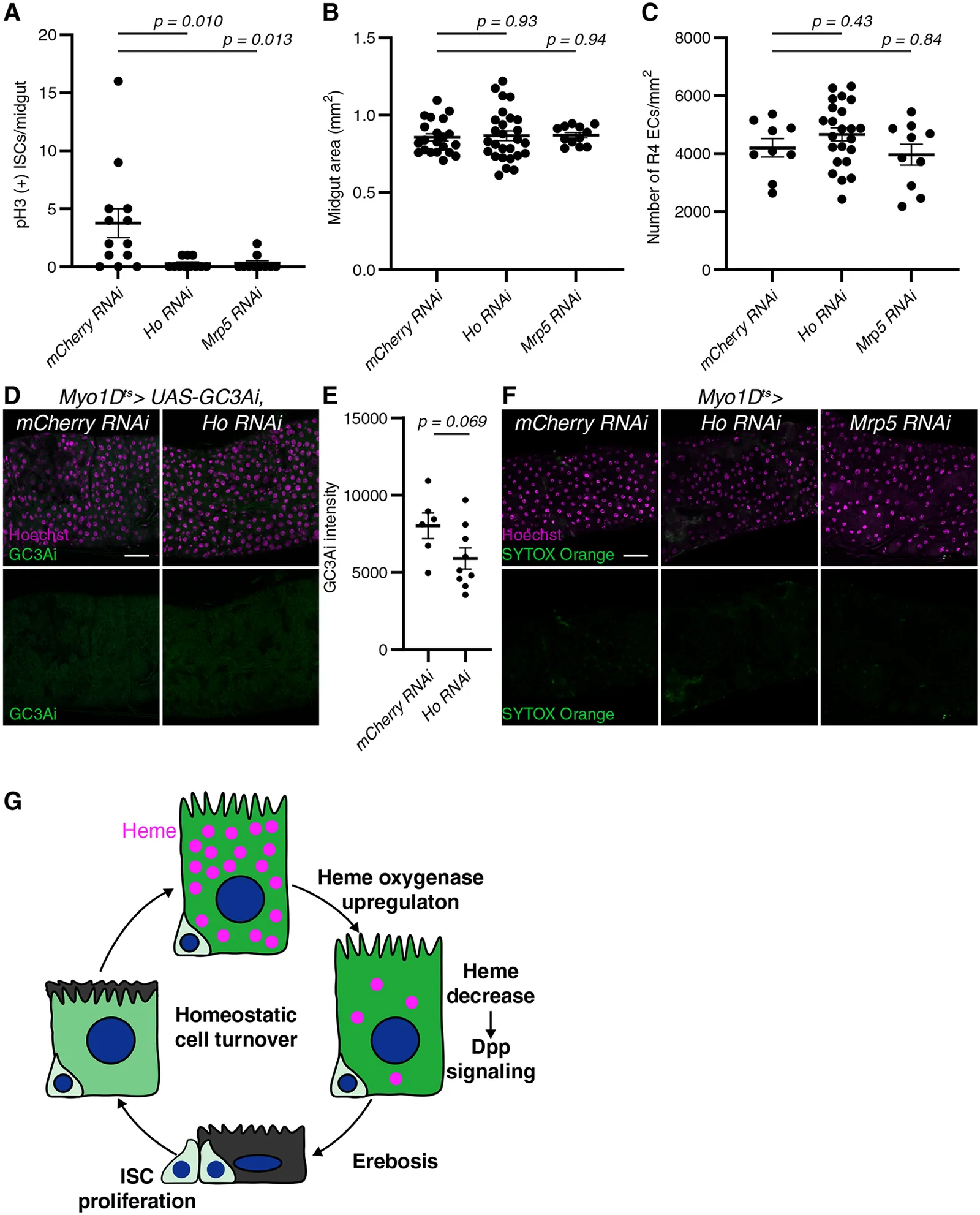
Reduction of erebosis is compensated by decreased ISC proliferation. **(A)** Inhibition of erebosis reduces ISC proliferation. *Ho RNAi* or *Mrp5 RNAi* significantly decreases a mitosis marker phopho-histone H3 (pH3)+ ISCs. **(B, C)** Inhibition of erebosis does not change the gut size. *Ho RNAi* or *Mrp5 RNAi* does not affect the gut area (B) or the density of enterocytes (C). **(D)** Heme inhibition does not induce apoptosis. *Ho RNAi* does not increase effector caspase sensor GC3Ai (green). **(E)** Quantification of (D). *Ho RNAi* does not increase GC3Ai signal compared with control *mCherry RNAi*. **(F)** Excess heme does not induce necrosis. *mCherry RNAi*, *Ho RNAi,* or *Mrp5 RNAi* does not result in SYTOX orange+ (green) necrotic cells. **(G)** Schematic illustration of erebosis-mediated cell turnover. *P* values were calculated using Dunnett’s test (A, B, C) or an unpaired two-tailed *t* test (E). Scale bars, 50 μm. S1 Data provides the data shown in (A–C, E).

Finally, we investigated the role for heme-mediated regulation of erebosis in tissue homeostasis. Tissue homeostasis is maintained through a balance between cell death and proliferation [52]. To investigate whether erebosis is involved in this balance, we examined the effect of erebosis inhibition on ISC division in the midgut. ISC proliferation was assessed by a mitosis marker anti phospho-Histone H3 (pH3) staining. Quantification revealed a significant decrease of pH3+ ISCs when erebosis was suppressed (Fig 6A). In control guts, we observed 4–5 pH3+ ISCs per gut, consistent with previous reports [5]. In contrast, inhibition of erebosis by *Ho RNAi* or *Mrp5 RNAi* resulted in a marked reduction of mitosis, with fewer than one pH3+ ISC per gut on average, indicating that the decrease in ISC division counteracts the reduction in erebosis. As a result of this balanced mechanism between erebosis and mitosis, inhibition of erebosis did not change the gut size or the cell density (Fig 6B, 6C). Heme accumulation or depletion did not cause apoptosis or necrosis, as assessed using the GC3Ai sensor [53] and SYTOX (Figs 6D–6F, S8A–S8C). These results indicate that changes in erebosis are compensated for by ISC proliferation, rather than by other types of cell death. From these results, we conclude that erebosis cooperates with ISC proliferation and plays a crucial role in tissue homeostasis, maintaining a constant gut size.

## Discussion

We found that heme decreases in erebotic cells, and experimentally induced heme reduction promotes erebosis. Conversely, heme accumulation inhibits erebosis. Downstream of heme, Dpp signaling suppresses erebosis. These findings indicate a causal relationship between intracellular heme levels and the initiation of erebosis via Dpp signaling. In pre-erebotic cells, upregulation of *Ho* and *Mrp5* occurs. Taken together, we propose that heme reduction in enterocytes is a critical initial step in erebosis (Fig 6G).

Heme and its metabolism are well known to regulate the cellular oxidative–reductive state or modulate protein activities as a co-factor. Labile heme itself is pro-oxidative, while heme catabolism produces both antioxidants, bilirubin and CO, and prooxidant Fe²^+^. Due to the opposing roles for heme metabolism in redox regulation, both upregulation and downregulation of Ho have been implicated in cell death. Suppression of Ho or excess heme can promote multiple types of cell death, including apoptosis [54], autophagic cell death [55], and necrotic cell death [56–60], mainly due to oxidative stress due to increase of labile heme and/or decrease of bilirubin. On the other hand, Ho activation has also been associated with necrosis and ferroptosis [61–64], which is mainly attributable to prooxidant function of Fe^2+^ released from heme. Several studies also showed that excess heme inhibits apoptosis [65–67]. In contrast to these previous reports, heme regulates erebosis without inducing oxidative stress, as its manipulation did not change ROS and lipid peroxide levels. Furthermore, erebosis does not have any feature of apoptosis, necrosis, or autophagy [19], which phenotypically contrasts with the previously reported cell death controlled by heme metabolism. Thus, the relationship between heme and erebosis is unique and different from previously characterized heme-associated cell death.

Regarding signaling upstream of heme, since *Ho* is upregulated in proteasome-high pre-erebotic cells (S5B Fig) and proteasome inhibition suppresses erebosis (S5C Fig), we speculate that proteasome-mediated protein degradation plays a key role. Proteasome inhibition is expected to have a broad range of effects, and thus it will be important in future studies to identify the E2 and E3 ubiquitin ligases and their substrates that regulate erebosis. Downstream of heme, we found that heme enhances Dpp signaling, which suppresses erebosis. Dpp was reported to play critical roles in gut physiology by affecting both enterocytes and ISCs [8,48–51]. Dpp ligands are secreted by trachea [49], enterocytes [50], hemocytes [51], and visceral muscles [48]. At least, Dpp ligand transcription is mildly increased by heme increase (S7A Fig). How heme activates Dpp signaling in enterocytes, whether by affecting only Dpp ligands or also their receptors and downstream signaling cascade, remains to be clarified. Since our data suggest that heme itself suppresses erebosis independent of its metabolism and cellular oxidative stress, its binding to some proteins should be a key mechanism. Proteomics approaches, which recently uncovered numerous heme-interacting proteins besides traditional heme binding proteins [68,69], would elucidate regulatory mechanisms of heme-mediated Dpp signaling. In addition to identifying the upstream mechanism by which heme regulates Dpp signaling, how downstream signals of Dpp mediate erebosis needs to be clarified in the future study.

Regarding the role for heme-mediated erebosis in tissue homeostasis, we discovered that inhibition of erebosis also reduces ISC proliferation. It suggests that there is a mechanism that couples ISC proliferation and enterocyte erebosis. We have previously shown that ISCs are located in close proximity to erebotic cells, which possibly reflects the communication between enterocytes and ISCs [19]. Similarly, previous research also suggested a cross-talk mechanism between enterocytes and ISCs [18,70]. How ISCs sense erebotic enterocytes, either directly or indirectly, remains to be determined. Considering that Dpp ligands are transcriptionally increased under the conditions that suppress erebosis (S7A Fig), and that Dpp can inhibit ISC proliferation in some context [8,48–51], a parsimonious hypothesis is that Dpp signaling simultaneously regulates erebosis and ISC behavior, thereby coupling enterocyte loss with ISC proliferation. In addition, bulk RNA-seq analysis of guts under heme-increased conditions demonstrates changes in multiple potentially mitogenic secreted factors, including Wingless, EGF ligands, *Drosophila* insulin like peptides (Dilps) and Unpaired (S9 Fig), raising the possibility that these pathways may also contribute to intricate communication between erebotic cells and ISCs. Beyond biochemical autocrine or paracrine mechanisms, tissue-level biophysical properties such as intercellular tension may also influence ISC activity. Defining these intercellular communication mechanisms will be an important direction for future study.

## Methods

### Fly husbandry

Flies were maintained as previously described [5]. They were kept on standard fly food containing 0.8% agar, 10% glucose, 4.5% corn flour, 3.72% dry yeast, 0.4% propionic acid, and 0.3% butyl p-hydroxybenzonate. For RNAi induction with Gal80ts, crosses were performed at 18 °C and the resulting progeny were shifted to 30 °C.

### Drosophila stocks

The following stocks were used in this study.

Oregon R
w; Myo1D-Gal4; tub-gal80^ts^
w; Myo1D-Gal4, UAS-GFP, UAS-nlsRFP; tub-gal80^ts^
hsFLP; act>>Gal4; UAS-GFP
UAS-GC3Ai [53]
mCherry RNAi (BDSC 35785)
rpn1 RNAi (BDSC 34348)
rpn7 RNAi (BDSC 34787)
rpn11 RNAi (BDSC 3362)
pomp RNAi (BDSC 64914)
Ho RNAi (VDRC 330474)
Ho RNAi (VDRC 330568)
Mrp5 RNAi (BDSC 32842)
Mrp5 RNAi (NIG 4562R-1)
UAS-Mrp5 (BDSC 16948)
Cox10 RNAi (BDSC 51864)
Cchl RNAi (BDSC 65123)
Tkv RNAi (VDRC 105843)
Put RNAi (NIG 7904R-2)
Med RNAi (VDRC 106767)
UAS-Dpp (BDSC 1486)
UAS-Tkv^CA^ (BDSC 36536)
UAS-p35 (BDSC5073)
UAS-STAT92E^CA^ [71] (A gift from Dr. Tatsushi Igaki)

### Single-cell collection

Single cells were microsurgically isolated by micromanipulation. Midgut tissues were transferred into PBS and roughly dissected using the LYKOS laser system (Hamilton Thorne) in a micromanipulation chamber, which was mounted on a heated stage (37 °C) of an inverted microscope (Olympus). After cutting the midgut cell cluster, normal enterocytes and erebotic cells were distinguished. Cells were selected based on fluorescence signal, with GFP(+)/RFP(+) cells corresponding to normal enterocytes and GFP(−)/RFP(+) cells corresponding to erebotic cells. Selected cells were collected using a fire-polished injection pipette (inner diameter, 10 µm). Each isolated cell was washed twice in PBS and transferred in 0.5 µl PBS into an 8-well tube containing 1.5 µl cell lysis buffer (1.33 U/μl RNasin Plus (Promega), 13.33% RealTime ready Cell Lysis Buffer (Roche), 0.4% NP-40, and RNase-free water), overlaid with 5 µl Vapor-Lock Oil (Qiagen).

### RamDA-seq library preparation

Library preparation for RamDA-seq was performed as previously described [24] with the following modifications. All reactions up to second-strand synthesis were carried out at twice the reaction volume of the original protocol. In the RT-RamDA reaction, SIRV-Set 4 (Iso Mix E0, ERCC, and long SIRVs; Lexogen) was added at a ratio of 3:250,000. To adapt the protocol for Drosophila cells, 0.016 U/μL XRN-1 (NEB M0338) was added to the genomic DNA digestion mix to suppress rRNA-derived cDNA synthesis, and the reaction was incubated at 30 °C for 5 min. Additionally, the not-so-random primers were replaced with N6 random hexamers in both RT-RamDA and second-strand synthesis. After second-strand synthesis, 18 μL of AMPure XP beads (Beckman Coulter), diluted to reduce the bead concentration to 40%, was added to 10 μL of the product for purification. Dried beads were eluted with 3.75 μL of 2/3× diluted Tagment DNA Buffer (Illumina) to recover double-stranded cDNA. Libraries were prepared using one-quarter of the standard reaction volume according to the Nextera XT DNA Library Preparation Kit (Illumina) protocol. PCR amplification was performed for 19 cycles, and the products were purified using 1.2× AMPure XP beads and eluted in 24 μL of TE buffer. Libraries were quantified using MultiNA, pooled in equimolar amounts, and the final pooled library was adjusted to 2.8 pM for sequencing. Sequencing was performed on a NextSeq 500 using the Mid Output Kit v2.5 with 76-cycle paired-end reads.

### RNA sequencing data analysis

RamDA-seq reads were adapter- and quality-trimmed with fastq-mcf (ea-utils v1.1.2) [M-1]. Trimmed reads were aligned to the *D. melanogaster* reference genome (UCSC dm6) using HISAT2 v2.2.0 [M-2]. The resulting BAM files were coordinate-sorted with samtools [M-3], and mapping/library quality was assessed with RSeQC v3.0.1 [M-4]. Gene-level counts were generated with featureCounts (Subread v2.0.1) [M-5] using the options “-t exon -g gene_id” and the RefSeq dm6 GTF annotation. We evaluated quality control metrics and excluded low-quality samples based on RNA integrity inferred from 5′–3′ gene-body coverage profiles and a genome mapping rate of ≤50%.

Seurat (v4.3.0) was used for variable feature selection (vst; 2,000 features), scaling, and principal component analysis (PCA) [M-6]. Read counts were converted to transcripts per million (TPM) on a per-cell basis and log-transformed. Cells were clustered by k-means (base R; stats::kmeans) using the first two principal component scores (k = 3). Cluster markers were identified with Seurat’s FindMarkers based on the k-means labels, and marker genes were subjected to GO enrichment using FlyEnrichr. For the analysis of proteasome-high cells, markers were identified with FindMarkers function comparing proteasome-high cells and others.

### RNAi screening

w-; Myo1D-Gal4, UAS-GFP, UAS-nlsRFP; tub-Gal80ts virgin female flies were crossed with male RNAi flies at 18 °C. Newly eclosed virgin female flies were collected and briefly kept at 18 °C, then transferred to 30 °C to induce RNAi expression for 7 days. At 30 °C, flies were flipped to new vials every day. After the induction, guts were dissected in 1x PBS and fixed for 60 min at RT in PBS with 4% paraformaldehyde (PFA, Thermo Fisher Scientific, 43368). Fixed guts were washed in PBSTx (1x PBS with 0.1% Triton X-100) 3 times and incubated with PBSTx 100 μg/mL Hoechst 33342 (Thermo Fisher Scientific H1399) at 4 °C overnight. After the staining, guts were washed in PBSTx, mounted, and observed with a confocal microscope (Zeiss LSM900).

### Quantification of RNAi efficiency and cell signaling gene expression

Total RNA was purified with Maxwell RSC simplyRNA Tissue Kit (Promega) from Myo1D^ts^ > RNAi guts following the manufacturer’s instructions. Each RNA sample was from 5 guts, and 5 samples were used for one genotype. RNA samples were sequenced by NovaSeq 6000, and in total 405 M reads were obtained. Sequencing data were analyzed by CLC Genomics Workbench. RNAi efficiency was calculated from TPM values of control and RNAi samples. For the cell signaling genes, two *Ho RNAi*s and two *Mrp5 RNAi*s were combined, respectively, to minimize the off-target effects. Expression was calculated from TPM values.

### 5-ALA feeding

Flies were fed with standard food with or without 10 mM 5-ALA (FUJIFILM Wako) at 30 °C for 7 days.

### Immunohistochemistry

Guts were dissected in 1x PBS, fixed for 60 min at RT in PBS with 4% PFA, and washed by PBSTx for 3 times. They were incubated with PBSTx with 10% normal goat serum (NGS) and primary antibody at 4 °C overnight, followed by washing in PBSTx 3 times and incubation with PBSTx with 10% NGS, 100 μg/mL Hoechst 33342 and secondary antibody at 4 °C overnight. After the incubation, the guts were washed in PBSTx 5 times, mounted and observed. Antibodies and their dilutions are as follows:

Mouse anti-Multi Ubiquitin 1:200 (MBL D058-3)
Rabbit anti-Ance 1:1000 (A gift from Dr. Elwyn Isaac)
Rabbit anti-phospho-Histone3 1:200 (Merck 06-570)
Goat anti-rabbit IgG Alexa Fluor 568 1:500 (Thermo Fisher Scientific A11036)
Goat anti-rabbit IgG Alexa Fluor 633 1:500 (Thermo Fisher Scientific A21070)
Goat anti-mouse IgG Alexa Fluor 633 1:500 (Thermo Fisher Scientific A21052)

### H-FluNox staining

Guts were dissected in 1x PBS and incubated with PBS with 10 μM H-FluNox and 100 μg/mL Hoechst 33342 at RT for 30 min. After the staining, the guts were washed twice in PBS for 5 min, mounted and observed. The mean signal intensity was calculated using Fiji following the manual selection of cells of interest.

### Rhodamine feeding

Virgin female flies were fed with 1x SY food (0.8% agar, 5% sucrose 10% dry yeast, 0.4% propionic acid, and 0.3% butyl p-hydroxybenzonate) for 4 days followed by 1 day feeding of Rhodamine food (0.8% agar, 12.5% sucrose, 0.35% dry yeast, 0.4% propionic acid, 0.3% butyl p-hydroxybenzonate, and 0.1% Rhodamine B) as previously described [43]. Flies were flipped to new vials every day. Guts were dissected in PBS, stained by PBS with 100 μg/mL Hoechst 33342 for 30 min at RT, washed in PBS, mounted and observed.

### CYP P450 inhibitor feeding

Flies were fed with standard food with 15 μM 1-ABT (MedChemExpress HY-103389) or PBO (MedChemExpress HY-B1198) at 30 °C for 7 days.

### DHE staining

hsFLP; act>>Gal4; UAS-GFP, RNAi adult virgin female flies were incubated at 37 °C for 30 min to induce act-Gal4 > RNAi clones. Animals were reared for 7 days with flipping to new vials every 2 days. Guts were dissected in 1x PBS and incubated with PBS with 15 μM dihydroehidium (DHE, Fujifilm Wako, 041-28251) and 100 μg/mL Hoechst 33342 at RT for 5 min. After the staining, the guts were washed in PBS, mounted and observed.

### Liperfluo staining

Guts were dissected in 1x PBS and incubated with PBS with 1 μM Liperfluo (Dojindo L248) and 100 μg/mL Hoechst 33342 at RT for 30 min. After the staining, the guts were washed in PBS, mounted and observed.

### RNAscope

RNAscope multiplex FL v2 (Advanced Cell Diagnostics) was conducted following the published protocol [72] and the manufacturer’s protocol with a slight modification. Guts were dissected in 1x PBS, fixed for 45 min at RT in PBS with 4% PFA, and washed by PBT (1x PBS with 0.3% Triton X-100) for 3 times. They were dehydrated by the sequence of PBT with 30, 50, and 70% methanol, followed by 100% methanol. After the dehydration, samples were rehydrated by the sequence of PBT with 70, 50, and 30% methanol, and washed by PBT for 5 min. Rehydrated samples were incubated with RNAscope Protease III reagent at 40 °C for 5 min, followed by PBT wash for 2 times and a post-fixation by PBS with 4% PFA, and PBT wash for 3 times. Post-fixed samples were hybridized with RNAscope probe at 40 °C for overnight, followed by AMP1, 2, 3, and HRP-C1, following the manufacturer’s instructions. After the hybridization, samples were incubated with Opal 570 dye (AKOYA Bioscience, 1:1500), washed with RNAscope wash buffer, stained with DAPI, mounted, and observed.

### Analysis of the gut size

To obtain the midgut area size, Myo1D^ts^ > GFP, nlsRFP, RNAi guts were observed and imaged using a fluorescent microscope (SMZ18, Nikon). The size of the Myo1D-driven GFP and nlsRFP positive area was calculated using Fiji. For the analysis of enterocyte density, the number of enterocytes (cells with large nuclei) and the size of the imaged tissue area were measured with Fiji following the imaging of R4 region.

### Statistics

Statistical analyses were conducted by R version 4.1.2. For multiple comparisons, *p*-values were corrected by Benjamini–Hochberg method. Error bars indicate standard errors.

## Supporting information

S1 Fig Quality control of single-cell RNA-seq data. **(A)** Isolation and single-cell RNA-seq analysis of normal enterocytes and erebotic cells. GFP high normal enterocytes and GFP low erebotic cells were manually collected, followed by sequencing and transcriptomic analysis. **(B)** Genome mapping rate (%) per cell before and after filtering out low-quality cells. **(C)** Number of detected genes per cell before and after filtering. **(D)** Gene body coverage profiles before and after filtering, showing normalized read coverage along the 5′–3′ gene body. **(E)** The result of the clustering analysis. Enterocytes and erebotic cells are divided into three clusters: normal cell cluster, erebotic cell cluster, and damaged cell cluster. S1 Data provides the data shown in (B–E).
(TIF)

S2 Fig Identification of erebotic cell cluster and damaged cell cluster. **(A)** Differentially expressed gene analysis reveals that *CG8745* and *LManIII* are markers of erebotic cell cluster, and *CG33926* is a marker of normal cell cluster. **(B)** RNAscope (in situ hybridization) reveals expression of marker genes shown in (A). *CG8745* or *LManIII* (magenta in cytoplasm) signals are positive in GFP (−) erebotic cells (arrows). In contrast, *CG33926* is expressed in GFP+ enterocytes (arrowheads) but not in GFP− erebotic cells (asterisks). Note that several GFP (+) cells (arrowheads) also express *CG8745* or *LManIII*, consistent with the clustering analysis in S1E Fig. Scale bar, 25 μm. **(C)** GO analysis of the damaged cell cluster. **(D)** STAT activation does not induce erebosis. Expression of constitutively active STAT92E does not increase GFP (−) nlsRFP (+) erebotic cells compared with control *mCherry RNAi*. Scale bar, 50 μm. S1 Data provides the data shown in (A, C).
(TIF)

S3 Fig Validation of scRNA-seq clustering on Fly Cell Atlas dataset. **(A)** tSNE plot of enterocyte clusters from Fly Cell Atlas gut_10x_stringent dataset. **(B)** Module score of R4-specific genes. Enterocyte (EC) of posterior adult midgut epithelium and Midgut large flat cell clusters show the highest scores. **(C)** Violin plot of the R4 module scores shown in (B). The scores are significantly higher in EC of posterior adult midgut epithelium and Midgut large flat cell clusters compared with the combination of all enterocyte clusters. **(D–F)** Module scores of top20 enriched genes in (D) normal cell cluster, (E) erebotic cell cluster, and (F) damaged cell cluster in EC of posterior adult midgut epithelium and Midgut large flat cell clusters. (D) Normal cell cluster score is higher in the upper region (dashed circle). (E) Erebotic cell cluster scores higher in the bottom region (dashed circle). (F) Damaged cell cluster score does not show a clear enrichment. **(G)** Scatter plot of normal cell cluster score and erebotic cell cluster score in cells from EC of posterior adult midgut epithelium and Midgut large flat cell clusters. Normal cell cluster score-high cells (red circle) and erebotic cell cluster score-high cells (red dashed circle) do not overlap. *P*-value was calculated using Dunnett’s test (C). S1 Data provides the data shown in (A–G).
(TIF)

S4 Fig List of top 20 genes of average log2 fold change. The top 20 genes with the most pronounced expression changes in each cluster are shown. S1 Data provides the data shown in the graphs.
(TIF)

S5 Fig Transcriptomics and RNAi screening for erebosis regulators. **(A)** Gene ontology (GO) analysis of the normal cell cluster shown in S1E Fig. Proteasome Degradation pathway is most strongly upregulated. **(B)** Expression heatmap of 26S proteasome genes and heme-related genes (*Ho* and *Mrp5* on the rightmost) in normal cell cluster. Each row represents the expression in one cell. Three cells at the bottom up-regulate proteasome genes and heme-related genes. **(C)** RNAis for *rpn1*, *rpn7*, *rpn11,* or *pomp*, all of which are proteasome components reduce the number of GFP− erebotic cells, while control *mCherry RNAi* gut has many such cells. Polyubiquitin signals are promoted in *rpn7 RNAi* gut, indicating that it reduces degradation of ubiquitinated proteins. Scale bars, 50 μm. S1 Data provides the data shown in (A and B).
(TIF)

S6 Fig RNAis for *Ho* and *Mrp5.* **(A, B)** TPM values of *Ho RNAi* (A) and *Mrp5 RNAi* (B) relative to control mCherry RNAi. **(C)** Apoptosis inhibition does not restore the erebosis inhibition by *Ho RNAi* or erebosis induction by *Mrp5* overexpression. **(D)** Classification of (C). p35 overexpression does not modify erebosis levels in *Ho RNAi* (left) or *Mrp5* overexpression (right). Scale bar, 50 μm. *P* values were calculated using likelihood ratio test (A, B) or Fisher’s exact test (D) and adjusted with the Benjamini–Hochberg method (A, B). S1 Data provides the data shown in (A, B).
(TIF)

S7 Fig Dpp signaling in erebosis. **(A)** RNA-seq of the whole midgut shows that erebosis inhibition by *Ho RNAi* or *Mrp5 RNAi* mildly increases Dpp ligands *dpp* and *gbb*. **(B)** Apoptosis inhibition does not restore the erebosis induction by *Tkv RNAi* or *Med RNAi*. **(C)** Classification of (B). p35 overexpression does not modify erebosis levels in *Tkv RNAi* (left) or *Med RNAi* (right). Scale bar, 50 μm. *P*-values were calculated using likelihood ratio test (A) or Fisher’s exact test (C) and adjusted with the Benjamini–Hochberg method (A). S1 Data provides the data shown in (A).
(TIF)

S8 Fig Heme depletion does not increase apoptosis or necrosis. **(A)** Heme depletion does not induce apoptosis. *UAS-Mrp5* does not increase effector caspase sensor GC3Ai (green). **(B)** Quantification of **(A)**. *UAS-Mrp5* does not increase GC3Ai signal compared with control *mCherry RNAi*. **(C)** Heme depletion does not induce necrosis. *mCherry RNAi* or *UAS-Mrp5* do not result in SYTOX orange+ (green) necrotic cells. *P*-value was calculated using unpaired two-tailed *t* test (B). Scale bars, 50 μm. S1 Data provides the data shown in (B).
(TIF)

S9 Fig Erebosis reduction changes signaling ligand expression. RNA-seq of the whole midgut shows that erebosis inhibition by *Ho RNAi* or *Mrp5 RNAi* reduces expression of several signaling ligands including *wg*, while increasing expression of other ones including *Krn*. Dotted lines indicate the average expression levels in control. *P*-values were calculated using likelihood ratio test and adjusted with the Benjamini–Hochberg method. S1 Data provides the data shown in the graph.
(TIF)

S1 Data Source data. The numerical values used for all main and supporting figures are provided.
(XLSX)

## Abbreviations

1-ABT: 1-Aminobenzotriazole
5-ALA: 5-aminolevulinic acid
CO: carbon monoxide
DEGs: differentially expressed genes
DHE: dihydroethidium
GO: gene ontology
*Ho*: *Heme oxygenase*
ISCs: intestinal stem cells
NAC: N-acetylcysteine
NGS: normal goat serum
PCA: principal component analysis
pH3: phopho-histone H3
pMad: phosphorylated Mad
PBO: piperonylbutoxide
PFA: paraformaldehyde
ROS: reactive oxygen species
TPM: transcripts per million.
